# Shattering and yield expression of sesame (*Sesamum indicum* L) genotypes influenced by paclobutrazol concentration under rainfed conditions of Pothwar

**DOI:** 10.1186/s12870-023-04145-7

**Published:** 2023-03-13

**Authors:** Jahangir Ahmed, Ghulam Qadir, Muhammad Ansar, Fahad Masoud Wattoo, Talha Javed, Baber Ali, Romina Alina Marc, Mehdi Rahimi

**Affiliations:** 1 Department of Agronomy, PMAS – Arid Agriculture University Rawalpindi, Rawalpindi, 46300 Pakistan; 2 Department of Plant Breeding & Genetics, PMAS – Arid Agriculture University Rawalpindi, Rawalpindi, 46300 Pakistan; 3 Department of Agronomy, University of Agriculture, Faisalabad, 38040 Pakistan; 4 Department of Plant Sciences, Quaid-i-Azam University, Islamabad, 45320 Pakistan; 5 Food Engineering Department, Faculty of Food Science and Technology, University of Agricultural Science and Veterinary Medicine Cluj-Napoca, 3-5 CaleaMănă̧stur Street, 400372 Cluj-Napoca, Romania; 6 Department of Biotechnology, Institute of Science and High Technology and Environmental Sciences, Graduate University of Advanced Technology, Kerman, Iran

**Keywords:** Sesame, Rainfed, Paclobutrazol, Shattering, Seed yield

## Abstract

Seed shattering is a critical challenge that significantly reduces sesame production by 50%. These shattering losses can be reduced by selecting shattering resistant genotypes or by incorporating modern agronomic management such as paclobutrazol, which can boost productivity and prevent seed shattering in sesame. Two-years of field trials were conducted to examine the effect of sesame genotypes, environment, and paclobutrazol (PBZ) concentrations. Twelve sesame genotypes were used in a four-way factorial RCBD with three replications and five PBZ concentrations (T_0_ = Control; T_1_ = 150; T_2_ = 300; T_3_ = 450; and T_4_ = 600 mg L^− 1^) under rainfed conditions of Pothwar. The findings revealed significant variations in the major effects of all examined variables (genotypes, locations, years, and PBZ levels). Sesame genotypes PI-154304 and PI-175907 had the highest plant height, number of capsule plant^− 1^, seed capsule^− 1^, 1000 seed weight, biological yield, and seed yield, while also having the lowest seed losses and shattering percentage. Regarding environments, NARC-Islamabad generated the highest plant height, number of capsule plant^− 1^, shattering percentage, and biological yield; however, the URF-Koont produced the highest seed yield with the lowest shattering percentage. Additionally, plant height, capsules plant^− 1^, and biological yield were higher in 2021, while seed capsule^− 1^, 1000 seed weight, seed losses, shattering percentage, and seed yield were higher in 2020. PBZ concentration affected all measured parameters; plant height and number of seed capsule^− 1^ decreased with increasing PBZ concentrations. 450 mg L^− 1^ PBZ concentration generated the highest biomass, number of capsules plant^− 1^, and seed yield. At the same time, PBZ concentration 600 mg L^− 1^ generated the smallest plant, the lowest seed capsules^− 1^, the greatest thousand seed weight, and the lowest shattering percentage. The study concluded that paclobutrazol could dramatically reduce shattering percentage and shattering losses while increasing economic returns through better productivity. Based on the findings, the genotypes PI-154304 and PI-175907 with paclobutrazol level 450 mgL^− 1^ may be suggested for cultivation in Pothwar farming community under rainfed conditions, as they showed promising shattering resistance as well as enhanced growth and yield.

## Introduction

Sesame (*Sesamum indicum* L) is associated with the Pedaliaceae family, which is presently considered an orphan crop in Pakistan. The life cycle of Sesame is typically 70-150 days long, depending on the variety and climate [1]. Its nutritional quality is of prime importance for humans containing 40-60% oil and 17-29% protein contents. Traditionally, sesame is the most significant oilseed crop in locations with scarce soil and drought [2–4]. Depending on the cultivar, it is one of the highest oil-comprising oilseed crops. Oil is extracted from sesame seeds worldwide, most of which is used for cooking and as a culinary oil. Sesame seed oil is odorless, edible, stable, and resistant to the oxidative deterioration. The composition of sesame fatty acid and oil contentssesame is mostly determined by prevailing environmental conditions. Oil quality for human consumption is determined by the concentration of UFAs (unsaturated fatty acids) and PUFAs (polyunsaturated fatty acids).

Pakistan suffers from a severe scarcity of edible oil production. In 2020-2021 (July-March), the countries edible oil demand of country was 3.291 million tonnes, with domestic production of 0.374 million tonnes (11.4%) and imports of 2.917 million tonnes (88.6%) contributing to the remainder. As a result, more local oilseed production is required. The 1.82 million-hectare Pothwar plateau, located between latitude 32°10 and 3°49 N and longitude 71°10 to 73°55 E, is an essential section of Pakistan subtropical dry land zone of Pakistan. The region experiences a wide variation of rainfall, with 400 mm in the southwest and 1500 mm in the northeast [5, 6]. Pothwar receives more than 70% of its annual precipitation during the summer, resulting in a semi-arid to humid climate [7]. In rainfed areas, biotic [8–10] and abiotic stresses [7, 11–24] have a negative impact on sesame yield and its components.

Another crucial element that significantly reduces sesame production is seed shattering. Shattering is the seed loss from shattered capsules before or during harvesting. Internal and external stresses, contact between plant components or harvest machinery, and changes in temperature [25], humidity, and capsule moisture are all factors that lead to shattering losses [26, 27]. However, these losses can be reduced by selecting shatter-resistant cultivars or implementing novel agronomic management strategies, such as using plant growth regulators [28] and micronutrients [29]. Several plant growth regulators have been used successfully to control plant growth and development, including paclobutrazol, mepiquat chloride, and chlorocholine chloride [30]. Previous research has also indicated that Paclobutrazol treatment minimizes shattering losses in shatter-prone crops [31, 32]. Furthermore, paclobutrazol has been used successfully to boost production and manage seed cracking in Birds-foot-trefoil [33, 34] and canola [35].

Paclobutrazol is a triazole-based chemical used to influence growth and physiological activities in several plant species. Paclobutrazol regulates plant development and physiological function by inhibiting the manufacture of sterol and gibberellic acid [36, 37]. Paclobutrazol prevents the oxidation of ent-kaurene to ent-kauronoic acid by inactivating cytochrome-dependent oxygenase [38–41]. As a result, paclobutrazol could be used as a stress protectant to manage plant water relations (such as capsule wetness) and breaking under stress conditions. The previous study has shown that using paclobutrazol at the proper concentration can considerably influence morphological and growth responses in plants, as well as boost seed output and shattering resistance [35, 38, 42, 43]. Paclobutrazol use, on the other hand, significantly reduced agricultural input [44, 45]. As a result, the effects of paclobutrazol administration on plant growth characteristics, seed yield, and shatter resistance may be erratic since they are linked to a variety of other factors such as meteorological circumstances, management tactics, and plant responsiveness [46, 47].

However, in Pothwar rainfed environment, choosing uniform capsule maturation and shatter-free genotypes, as well as better agronomic methods, significantly increased crop seed output [48, 49]. As a result, a thorough investigation was required to determine the optimum dose of paclobutrazol for improved sesame production, particularly under rainfed circumstances. As a result, a two-year field study was launched to further characterize sesame responses to paclobutrazol treatment. The primary goals of this study were to optimize sesame production technology and to assess the effects of various paclobutrazol concentrations on the yield and shattering of sesame genotypes under rainfed circumstances in Pothwar.

## Materials and methods

### Field experimentation site and treatments description

Experiments were conducted over 2 years at two locations, viz., the University Research Farm (URF) Chakwal Road in Rawalpindi (elevation, 510 m; latitude 33.1° N; longitude 73.0° E; annually rainfallbetween 650–850 mm); and the National Agricultural Research Center, Islamabad (NARC) with 504 m elevation; 33.7° N latitude; 73.1° E longitude; 1000-1200 mm of annual rainfall. The experiments were carried out in 2020 & 2021 at each location. In this experiment, the ten sesame genotypes were acquired from the Plant Genetic Resources Conservation Unit (USDA) Griffin Georgia United States, and in a couple of indigenous varieties from the Ayub Agriculture Research Institute Faisalabad (PI-154304, PI-158053, PI-164763, PI-158902, PI-158918, PI-164142, PI-164317, PI-164732, PI-153509, PI-175907, TS-3, and TH-6) originated from different countries (Table 1). Discover very promising sesame genotypes with high yielding and shattering resistance under rainfed conditions of Pothwar. It is planned to use a four-way factorial randomized complete block (RCBD) design with three replications and five different paclobutrazol doses (T_0_ = Control without paclobutrazol, T_1_ = 150, T_2_ = 300, T_3_ = 450, and T_4_ = 600 mg L^− 1^ ha^− 1^).

**Table 1:** Origin of sesame genotypes

Genotypes No	Origin
PI-153509	Venezuela
PI-154304	Mexico Sonora
PI-158053	China
PI-158902	China
PI-158918	China
PI-164142	India
PI-164317	India Tamilnadu
PI-164732	India
PI-164763	India
PI-175907	Turkey
TH-6	AARI Faisabad (Pakistan)
TS-3	AARI Faisabad (Pakistan)

The genotypes were grown in June, 2020 and 2021, respectively. The seeds were seeded using the manual drill method, with each genotype having a four-meter length, and the plant-to-plant distance was kept at 20 cm, while the row-to-row space was held at 45 cm. With a single-row hand drill, crops were planted in 2.7 m × 4 m plots with six rows in each field. The 3 kg ha^− 1^ seed rate and the approximately 3 - 4 cm seeding depth were kept. Forty days after sowing (40 DAS) was targeted for application of paclobutrazol concentrations as a foliar application. All cultural and agronomic procedures were applied uniformly across all treatments in both sites. Manual thinning was undertaken after the entire emergence to preserve the space between plants. Each plot was responsible for recording the following observation. Ten plants were chosen randomly for data collection based on the previously descriptors provided by the (Langham 2007 & 2016), and the following measurements related to high yielding and shattering resistance were investigated.

### Soil and meteorological conditions

**Fig. 1:**
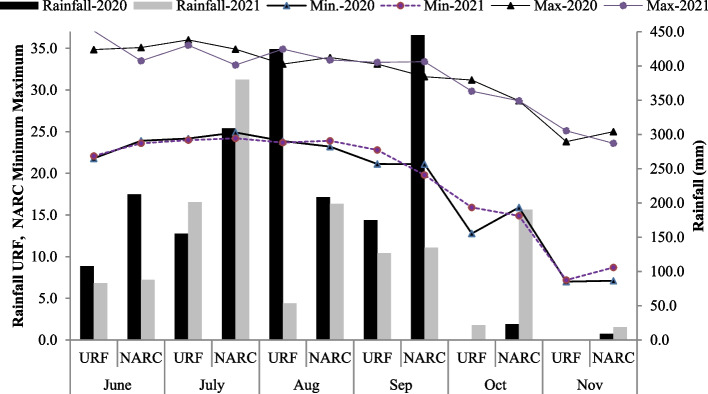
Monthly temperature minimum (Min), maximum (Max) and rainfall during the growing season of 2020 and 2021

Pre-sowing composite soil samples (0-15 cm) from both locations were collected and evaluated for soil physicochemical parameters (Table 2). In both years, meteorological conditions intended for the research spots were acquired from Pakistan Pakistan Meteorological Department in Islamabad (Fig. 1).

**Table 2:** Soil physiochemical assessment of the experimental locations during 2020 and 2021

Soil Characteristics	Units	NARC Field (Islamabad)	URF Koont Farm (Rawalpindi)
Sand	%	34	56
Silt	%	33	22
Clay	%	33	23
Ec	dS m^−1^	0.23	1.09
pH		8.0	8.2
CaCO_3_	%	5.15	5.23
K	mgkg^−1^	100	89
P	mgkg^−1^	4.1	3.3
NH_4_	mgkg^−1^	0.7	0.5
NO_3_	mgkg^−1^	5.3	4.2
Organic matter	%	0.98	0.65
Saturation percentage	%	32.6	29.5
Textural class		Silt Loam	Sandy Clay Loam

### Crop data

Sesame genotypes were evaluated for their growth, yield, and shattering characteristics. At maturity, plant height (measured with a tape) and the number of capsules per plant were collected from 10 randomly chosen plants in each plot, and the average per plant was calculated. One hundred ripe plant capsules were sampled randomly from each plot to acquire information regarding the number of seeds capsule^− 1^.Following manual threshing of the capsules, the total number of seeds was counted using a seed counter, and the mean number of seeds capsule^− 1^ was computed.

The matured plants in the two middle rows of each plots plot were harvested by hand, and the bundles of plants were stored vertically in the field. After a few days of sun drying, the biological yield of each plant bundle was measured with a scale and converted to kg hectare^−1^. By eliminating plant debris after physically threshing the plant bundles, clean seeds were retrieved. Using an electric balance, the weight of each seed sample was recorded, and the seed yield in kg hectare-^1^ was calculated. From the bulk seed sample obtained from each plot, four batches of 1000 seeds were sorted using a seed counter, the weight of each batch was recorded using an electric balance, and the average weight of thousand seeds was calculated. Similarly, for shattered seed g per m^2^, the total seed losses per m^2^ in each treatment were recorded by an electronic balance in grams, and the entire seed losses were converted to kg ha^− 1^. Ten randomly selected plants from each treatment were evaluated for their resistance to sesame seed shattering. The shattering estimation was done by clipping pods at physiological maturity and sun drying for 10 days after harvest. In each plot total number of shattered and non-shattered pods was counted, and then the plant^− 1^ average was calculated [50, 51]. The shattering % was calculated by using the formula as under.

$$\text{Shattering }\%=\frac{\text{Total}\;\text{number}\;\text{of}\;\text{non-shattered}\;{\text{pods}\;\text{plant}}^{-1}}{\text{Total}\;\text{number}\;\text{of}\;\text{shattered}\;{\text{pods}\;\text{plant}}^{-1}}\times100$$

### Statistical analysis

A generalized-linear model was used for the analysis of variance (ANOVA) using statistics 8.1, where an RCB four-way factorial design was utilized to regulate the level of significance of the leading factors (genotypes, paclobutrazol concentration, locations, and years) and their potential interactions. Furthermore, the least significance test (LSD) was used with a probability level of (*P* ≤ 0.05) to discover significant variance between the mean. Moreover, Pearsons Pearsos correlation and principal component analysis between agronomic traits using R software to categorize linear associations between examined traits & sesame genotypes and to identify shattering resistant sesame genotypes were performed [52].

## Results

### Sesame genotypes

The primary impacts of sesame genotypes on studied growth, yield, and shattering attributes were determined to be highly significant at (*P* ≤ 0.05), as presented in Table 3. The genotype PI–154304 had the highest values for plant height, number of capsules plant^−1^, and biomass yield, which was statistically at par with PI-175907, PI-154304 and PI-153509. The highest seed yield (1575 kg ha^−1^) was produced by the genotype PI-175907, which was statistically parallel with PI-154304 (1564 kg ha^−1^) andgenotype PI-153509 (1560 kg ha^−1^) followed by the genotype PI-158918 (1181 kg ha^−1^). But these genotypes were lowest for seed losses (211.2, 220.7, and 237.6 kg ha^−1^) and shattering percentages (28.4, 28.7, and 29.4%), respectively.

**Table 3:** Main and interactive effects of sesame genotypes, paclobutrazol levels, location, and years for growth, shattering, and yield attributes

	Plant Height (cm)	No. of Capsules Plant^**−1**^	No. of Seeds Capsule^**−1**^	1000 Seeds Weight (g)	Seed losses (kg ha^**−1**^)	Shattering %	Biological Yield (kg ha^**−1**^)	Seed Yield (kg ha^**−1**^)
**Genotypes** (G)	***	***	***	***	***	***	***	***
PI – 153,509	123.8 a	226.0 a	66.1 b	5.2 ab	237.6 f	29.4 h	5016 a	1560a
PI – 154,304	128.1 a	227.5 a	68.5 ab	5.1 b	211.2 g	28.4 h	5092 a	1564 a
PI – 158,053	108.0 b	117.1 e	45.4 e	3.0 i	394.6 a	38.5 f	3845 de	1170 bc
PI – 158,902	83.0 e	148.9 d	63.2 c	3.1 i	364.3 b	45.5 c	3838 d-f	1124 b-d
PI – 158,918	82.1 e	179.8 c	47.5 e	4.0 f	388.2 a	50.8 b	4141 b	1181 b
PI – 164,142	95.5 d	90.7 fg	63.0 c	4.4 d	329.1 c	38.8 f	3910 c-e	1075 d
PI – 164,317	98.5 cd	95.9 f	62.2 c	3.8 g	299.6 de	35.9 g	3635 f	1108 cd
PI – 164,732	101.5 c	123.4 e	55.8 d	4.6 c	309.8 d	40.3 e	4076 bc	1178 b
PI – 164,763	102.1 c	188.8 b	37.0 f	3.3 h	287.9 e	42.4 d	3737 ef	1112 cd
PI – 175,907	125.0 a	228.7 a	70.3 a	5.3 a	220.7 g	28.7 h	5109 a	1575 a
TH – 6	101.3 c	47.2 h	29.7 g	4.4 d	339.3 c	34.4 g	3994 b-d	1149 bc
TS – 3	103.2 bc	86.5 g	38.1 f	4.2 e	352.5 b	56.1 a	3753 ef	1131 b-d
LSD (0.05%)	5.3673	7.11	2.6458	0.1597	12.649	1.5426	204.38	63.928
**Locations** (L)	***	***	***	***	***	**	***	***
NARC Islamabad	109.7 a	154.5 a	51.8 b	4.2 a	281.0 b	38.1 b	4630 a	1123 b
URF- Koont	98.9 b	138.9 b	56.0 a	4.2 a	341.5 a	40.1 a	3727 b	1365 a
LSD (0.05%)	2.1912	2.9027	1.0802	0.0652	5.1639	0.6298	83.437	26.099
**Year**	***	***	***	***	**	***	***	***
2020	100.7 b	142.2 b	55.7 a	4.4 a	314.5 a	40.1 a	4038 b	1287 a
2021	107.8 a	151.2 a	52.1 b	4.0 b	308.0 b	38.1 b	4320 a	1201 b
LSD (0.05%)	2.1912	2.9027	1.0802	0.0652	0.0139**	0.6298	83.437	26.099
**Paclobutrazol** (P)	***	***	***	***	***	***	***	***
Control	131.0 a	135.4 d	64.5 a	3.7 e	346.9 a	43.3 a	3799 d	1175 c
150 mg L^−1^	112.1 b	143.7 c	62.5 b	3.9 d	328.8 b	39.9 b	4128 c	1225 b
300 mg L^−1^	102.7 c	152.8 b	51.8 c	4.1 c	313.5 c	39.3 b	4287 b	1264 b
450 mg L^−1^	93.2 d	161.7 a	49.3 d	4.4 b	297.8 d	36.9 c	4602 a	1310 a
600 mg L^−1^	82.3 e	139.9 cd	41.4 e	4.8 a	269.3 e	36.0 c	4079 c	1245 b
LSD (0.05%)	3.4646	4.5895	1.7079	0.1031	8.1649	0.9957	131.93	41.266
G x L	**	NS	NS	NS	***	***	NS	NS
G x Y	NS	NS	NS	***	***	NS	NS	**
G x P	***	NS	NS	NS	***	***	NS	NS
L x Y	NS	NS	***	***	***	NS	NS	**
L x P	***	NS	NS	NS	***	***	**	NS
Y x P	NS	NS	NS	NS	NS	NS	NS	NS
G x L x Y	NS	NS	NS	NS	***	NS	NS	NS
G x L x P	***	NS	NS	NS	NS	NS	NS	NS
G x Y x P	NS	NS	NS	NS	NS	NS	NS	NS
L x Y x P	NS	NS	NS	NS	NS	NS	NS	NS
G x L x Y x P	NS	NS	NS	NS	NS	NS	NS	NS

*NS* Non-significant; * Significant; ** highly significant at *P* ≤ 0.05. Different letters indicate statistically significant – differences among the value in each column and individual factors (LSD test; *P* ≤ 0.05)

While the lowest yield was produced by the PI-164317 (1108 kg ha^−1^) due to minimum 1000 seed weight (3.8 g). In contrast, genotypes had a substantial effect on thousand seed weight and seed capsule^−1^. The more thousand seed weight were recorded in genotypes PI-175907 (5.3 g), followed by the sesame genotypes PI-153509 (5.2 g), and the less thousand seed weight were produced by the genotype PI-158053 (3.0 g).

The genotype PI-175907 has the most seed capsule^−1^ (70.3), followed by the genotype PI-154304 (68.5). The genotype TH-6 had the fewest seed capsule^−1^ (29.7). There is a lot of difference between sesame genotypes regarding growth, yield, and seed shattering [50, 53–56].

### Locations

There were statistically significant differences observed between the two Location means at (P ≤ 0.05) for all growth, yield, and shattering characteristics (Table 3). NARC, Islamabad produced more plants height (109.7 cm), number of capsule plant^−1^(154.5), shattering % (28.3%), and biological yield (4630 kg ha^−1^), and the URF Koont achieved the maximum seed yield of (1365 kg ha^−1^), with the shattering percentage (40.1%). Moreover, URF Koont and NARC Islamabad were statistically at par among locational means of 1000 seed weight. At the same time the minimum seed yield (1123 kg ha^−1^) was produced by the location NARC Islamabad.

### Year

There were statistically significant differences (*P* ≤ 0.05) between the year means for all the examined parameters. In the second year, 2021, the plant height was 107.8 cm, the number of capsules per plant was 151.2%, and the yield was 1201 kg ha^−1^(Table 3). However, seed capsule^−1^ (55.7), 1000 seed weight (4.4 g), seed losses (314.5 kg ha^−1^), shattering % (30.4%), and seed yield (1287 kg ha^−1^) were significantly higher than 2020.

### Paclobutrazol concentrations

Paclobutrazol concentration significantly affected all studied parameters at a significant level (*P* ≤ 0.05). Plant height, No. of capsules plant^−1^, seeds capsules^−1^, 1000 seed weight, seed losses, shattering percentage, total biomass, and seed yield of the sesame genotypes. A considerable difference was recorded for paclobutrazol treatments. Plant heights for the genotypes and paclobutrazol treatments have been presented in (Table 3). A significant reduction in plant height was observed with rising PBZ concentrations. Furthermore, the highest (131 cm) plant height was assessed for the control and the minimum (82 cm) for the P_6_ (600 mg L^−1^) treatment. However, the interaction (G x Y, L x Y, Y x P, G x L x Y, G x Y x T, and G x L x Y x T) was found non-significant. The numbers of capsules are the key determining factor of the economic yield in sesame. A significant increase in the number of capsules under a given area can substantially improve the economic yield of the crop. Statistical analysis revealed that a significant difference was computed for sesame genotypes and levels of paclobutrazol. A maximum number of capsules plant^−1^ (161.7) were counted in the P_4_ (450 mg L^−1^), while the minimum (135.4) capsules plant^−1^ were recorded in control (Table 3). While the interactions showed non-significant.

The number of seeds in capsule^−1^ significantly contributes to the overall seed yield of the cultivated crops. The paclobutrazol application significantly affected the number of seed capsule^−1^. The number of seeds in sesame showed a declining trend against the paclobutrazol concentrations (Table 3). Control depicted the highest (64.5) mean number of seeds in the capsule, whereas the P_5_ (600 mg L^−1^) treatment depicted the lowest (41.4) seeds capsule^−1^. In our study, there was a low number of seeds per capsule counted at higher paclobutrazol concentrations. However, all the interactions were found non-significant except (L x Y). Thousand grain weights significantly influence the final seed or grain yield of the crops. Significantly variation was observed amongst the paclobutrazol treatments (Control, 150, 300, 450, and 600 mg L^−1^). The highest (4.8 g) thousand grains were recorded in P_5_ (600 mg L^−1^) and minimum (3.7 g) in control. However, all the interactions except the (G x Y and L x Y) were found non-significant.

Significant variations were observed in the shattering losses for the paclobutrazol treatments. Moreover, among the paclobutrazol treatments, the maximum (346 kg ha^−1^) shattering losses were recorded in control while the minimum (269.3 kg ha^−1^) was recorded in P_5_ (600 mg L^−1^). However, the interaction (Y x P, G x L x P, G x Y x P, L x Y x P and G x L xY x P) were found non-significant. Increased shattering percentage reduces the total obtained yield of the sesame crop. Hence, the shattering percentage can significantly impact the crop yield at harvesting. The result indicates that shattering percentage can significantly be reduced by using paclobutrazol, and yield can be increased. A significant difference in shattering percentage has been observed for paclobutrazol treatments. The highest (43.3) percentage was observed in control, while the lowest (36%) was observed in P_5_ (600 mg L^−1^). While the interactions (G x L, G x P, and L x P) were found non-significant.

Total biomass is the overall accumulated dry matter of the crop. Biomass yield depends on several factors. The five paclobutrazol concentrations observed a significant difference in the sesame biological yield. The highest biomass (4602 kg ha^−1^) was recorded for paclobutrazol treatments P_4_ (450 mg L^−1^), whereas the minimum (3799 kg ha^−1^) was in control (Table 3). However, all the interactions are non-significant, and (L x P) were found significant. Grain yield considerably varied for the five paclobutrazol treatments (Control, 150, 300, 450, and 600 mg L^−1^). Moreover, for the paclobutrazol treatments, the lowest (1175 kg ha^−1^) grain yield was found in the standard treatment (control) even though the highest (1310 kg ha^−1^) in the P_4_ (450 mg L^−1^). However, all the interactions are non-significant, and (G x Y, L x Y) were found significant at *P* ≤ 0.05 (Table 3).

### Correlation studies for various agronomic characteristics of sesame

**Fig. 2:**
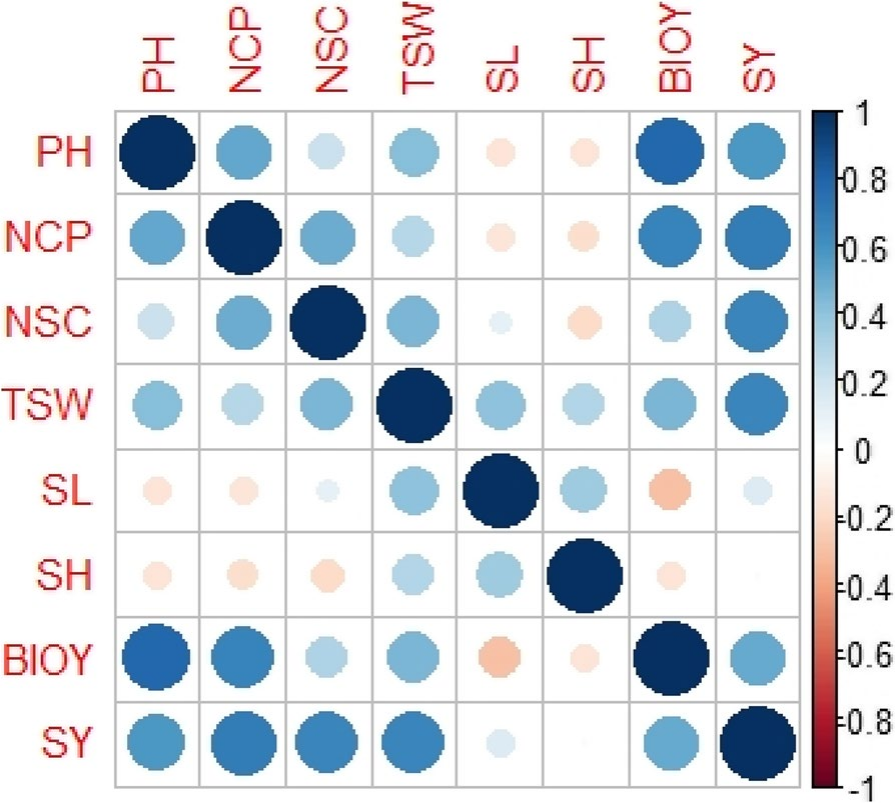
Correlation coefficients among the agronomic attributes of sesame genotypes

Plant height (PH), number of capsules plant^−1^ (NCP), number of seed capsule^−1^ (NSC), 1000 seed weight (TSW), and biomass yield all exhibited a highly significant and positive association with seed yield (Fig. 2). Negative associations were found between seed yield of sesame genotypes and shattering % (SH) and shattering losses (SL). Similarly, biomass yield was significant and favorably connected with NCP, NSC, PH, SL, and SY features while shattering percentage attributes were negatively correlated. Typically, yield and its attributing parameters contribute positively to ultimate seed yield nevertheless in this case, the sesame genotypes (PI-175907, PI-154304, and PI-153509) demonstrated some unique characteristics, such as NCP, NSC, and lower seed losses kg ha^−1^, as well as minimum shattering percentage, maximum biomass, and higher seed yield kg ha^−1^ when compared to the rest of the genotypes. Other growth and yield-related variables were thus adversely linked with PH, SL, NCP, NSC, and seed indices.

### Principal component analysis (PCA) for various agronomic characteristics of sesame

Principal component analysis (PCA) is a multivariate statistical technique based on trait correlation used to assess and simplify complex and large datasets to analyze genetic diversity and its relationship to observed qualities. The PC values explained the traits responsible for around 80% of the genotypic variability explained by the first three components. In contrast, the first two PCs explained 67% of the variability (Table 4). Individual performance variances were 44.98%, 22.5%, and 12.71% for PC1, PC2, and PC3, respectively. The first principal component (PC1) was characterized predominantly by positive contributions from PH, NCP, NSC, TSW, BIOY, and SY, whereas SL and SH have a devastating impact on the estimated variation. The second biggest principal component (PC2) was created to capture the wide variation from NSC, TSW, SL, SH, and SY. Similarly, NCP, NSC, SL, and SY positively contributed to PC3, while the remaining variables had low Eigen values in this principal component (Table 4).

**Table 4:** Factor Loadings, Eigen Values, and Individual & Cumulative Variation

	F1	F2	F3	F4	F5	F6	F7	F8
PH	0.7738	−0.2180	−0.3976	− 0.3052	0.0554	0.2166	0.2151	−0.0790
NCP	0.8040	−0.2090	0.1073	0.3261	0.4016	−0.1313	−0.0632	− 0.0977
NSC	0.6595	0.1496	0.6203	0.1377	−0.2782	−0.0450	0.2434	−0.0165
TSW	0.6818	0.5794	−0.1412	−0.1657	− 0.2771	−0.0997	− 0.2370	−0.0956
SL	−0.0158	0.8532	0.1514	−0.3253	0.3381	−0.1258	0.1062	0.0396
SH	−0.1168	0.6963	−0.5062	0.4732	−0.0416	0.0529	0.1294	0.0028
BIOY	0.8130	−0.3056	−0.3671	− 0.0280	−0.0740	− 0.2904	0.0456	0.1348
SY	0.8823	0.2111	0.1703	0.0783	0.0762	0.3128	−0.1528	0.1214
Eigenvalue	3.60	1.80	1.02	0.60	0.40	0.30	0.20	0.06
Variability (%)	44.98	22.50	12.71	7.28	5.57	3.46	2.74	0.75
Cumulative (%)	44.98	67.48	80.20	87.48	93.05	96.51	99.25	100.00

**Fig. 3:**
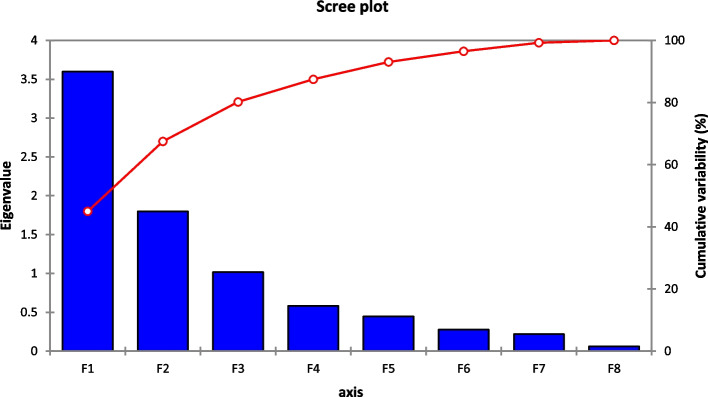
Scree Plot among the agronomic attributes of sesame genotypes

A PCA-based scree plot is a graph that indicates the eigen values and the principal component number. A total of eight principal components (PCs) were obtained in this study, although only three PCs having eigenvalues more extensive than one were well-thought-out significant, with the remaining PCs being non-significant. PC1 has an eigen value of 3.60, PC2 has a value of 1.80, and PC3 has a value of 1.02 (Fig. 3).

**Fig. 4:**
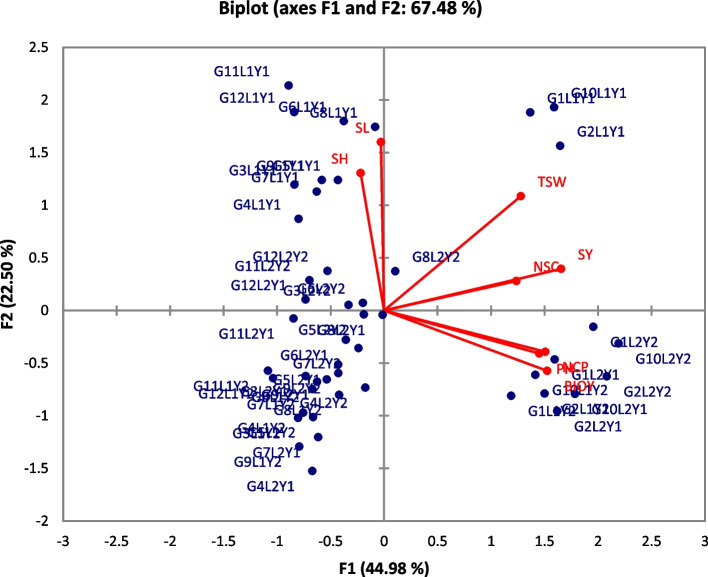
PCA Biplot for Agronomic Variables and Sesame Genotypes

A PCA biplot analysis can be used to find features that can be divided into primary groups and subgroups based on homogeneity and dissimilarity. The quantity and direction of loading vectors indicate a variables positive or negative contribution variables variables to total phenotypic diversity (Fig. 4). Furthermore, the essential features (SY, BIOY, TSW, NCP, PH, and NSC) that contribute significantly to the computed variance were assigned to the positive side of the loading plot. In contrast, left-aligned characters like SL and SH had a negative impact on overall diversity. The genotype distances showed the degree of difference between measured traits. For one or more criteria, the genotypes on either the positive or negative side of the biplot had comparable phenotypic performance (Fig. 4). Furthermore, the genetic distances between genotypes farthest from the origin were the highest. As a result, specific genotypes, PI – 175,907 (G_10_L_2_Y_2_), PI – 154,304 (G_2_L_2_Y_2_), PI – 154,304 (G_2_L_2_Y_1_), PI – 175,907 (G_10_L_1_Y_1_), PI – 158,902 (G_4_L_2_Y_1_), and TH – 6 (G_11_L_2_Y_1_), distributed at the score plots plot extreme places, exhibited the widest phenotypic variety. These genotypes may or may not be superior, but selection may consider them.

## Discussion

Sesame genotypes showed significantly different morphological attributes due to prevailing environmental factors and genetic variability. Similar results were reported in previous findings by Ahmed et al. [5], Khan et al. [54], Baraki et al. [57], Akhtar et al. [58]. As a result of their dominance in the genetics and yield stability of capsule plant^−1^, seed capsule^−1^, 1000 seeds weight and biological yield, PI-154304 and PI - 175,907 produced the highest seed yields among the number of plants. PI-153509 showed more capsule plants^−1^, seed capsules^−1^, and 1000 seed weight with low shattering percentage than the remaing genotypes, followed by the rest of the genotypes. But if produced under ideal conditions, these three genotypes have a somewhat higher yield potential than other genotypes. These three genotypes are highly drought and shattering resistant and reasonably tolerant to diseases and pests [54]. However, TS-3 produced the lowest seed yield due to a higher shattering percentage.

The findings are the pooled averages of sesame genotypes cultivated in two different sites with distinct climatic circumstances, especially the quantity and distribution of precipitation absorbed throughout the growing season. The genotypes of sesame differed between the two climatic conditions of Pothwar, particularly about the amount and distribution of rainfall throughout the crop growing season. In addition, the phenotypic expression of sesame genotypes varies in response to environmental and seasonal fluctuations [50, 54, 57]. Sesame genotypes performed differently regarding vegetative development and shattering percentage throughout two growing seasons. These findings are consistent with previous research [50, 54, 57]. The first-year cropping season precipitation was pretty high across all locations in the current study (Fig. 1). As a result, growth characteristics, particularly biological yield, were often better in the second year. This could be attributed to proper nutrient perception, good soil wetness, and suitable assimilation & translocation into the plants sections of plant, all of which could get enhanced photosynthetic rates and cause elevated vegetative expansion but decreased grain yield.

The unpredictable precipitation, soil aeration, and the genotypes physiological and morphological expression might all be blamed for the different effects of sesame genotypes in various climatic settings. On the other hand, sesame is particularly susceptible to drought during various morphological and physiological periods, although it is most susceptible to excessive water-logging and precipitation [55, 59, 60]. Excessive soil moisture reduces aeration and oxygen supply, reducing root respiration [61]. Because of a higher seed capsule^−1^, 1000 seed weight, and a lower incidence of shattering, the URF - Koont produced more seed than the NARC - Islamabad. Excess soil moisture caused by heavy rainfall in September 2020 and July 2021 (Fig. 1) may have increased growth characteristics while decreasing yield at NARC. Heavy rains can cause fertilizer leaching, resulting in lower sesame productivity. High soil moisture was found to cause a considerable drop in seed yield [54].

Plant growth regulators (Paclobutrazol) alter hormonal stability and development, resulting in enhanced seed production, improved crop tolerance to abiotic challenges, and enhanced physico-morphological properties of the crops [41, 50, 62, 63]. A considerable difference was recorded for paclobutrazol treatments. A significant reduction in plant height was observed by increasing PBZ concentrations. Furthermore, the highest plant height for the control and the minimum plant height for the P6 (600 mg L^−1^) treatment were observed. Similar results were reported in wheat, where the paclobutrazol application reduced the plant height significantly [44]. Our results were in line with the earlier reported results where a significant decrease in plant height was observed [35, 64]. Additionally, the reduction in plant height may be linked to the inhibition of gibberellin biosynthesis in plants [65, 66].

The number of capsules is key determining factor of the economic yield in sesame. An increase in the number of capsules per unit area can dramatically increase the economic production of crop.Additionally, a greater number of plant^−1^ capsules were tabulated in the P_4_ (450 mg L^−1^), while the minimum capsules plant^−1^ recorded in control. The retardation in apical growth at reproductive stage might lead to better assimilate partitioning in plant parts and ultimately increase the number of capsules [67]. Similar to the findings of other researchers, this study found that paclobutrazol increases the quantity of plant capsules [35, 53, 68]. The number of seeds capsule^−1^ substantially contribute to the overall seed yield of the cultivated crops. Paclobutrazol application significantly affected the number of seed capsule^−1^. Control depicted the highest mean number of seeds in capsule whereas the P_5_ (600 mg L^−1^) treatment depicted the lowest seeds capsule^−1^. In current study, there was insufficient number of seed capsule^−1^ were counted at higher paclobutrazol concentrations. Results in this study were consistent with the findings documented in the canola crop [35]. However, reducing the number of seeds in each capsule reduces competition for photosynthesis and nutrient allocation while increasing seed weight, which eventually increases seed output after paclobutrazol administration.

Thousand grain weights significantly influence the final seed or grain yield of the crop. The highest thousand grain were recorded in P_5_ (600 mg L^−1^) and minimum in control. The thousand grain weight was increased after paclobutrazol treatments. This increment in seed weight might be interlinked with the decreased seed number plant^−1^, which relieved the competition for the nutrients allocation among the seeds [69]. In present study, the results were in confirmation with the findings reported in canola crop [35].

Sesame is severely constrained by severe shattering before maturity and during harvesting. To improve economic returns, it is necessary to eliminate losses. Moreover, among the paclobutrazol treatments, the control had the highest level of shattering losses, whereas P_5_ had the lowest (600 mg L^−1^). Sesame is prone to severe shattering losses. Shattering losses at maturity is a major limitation in oilseeds (sesame, canola) production and mechanical harvesting [50, 70]. Shattering is caused due to environmental as well as genetic factors [51]. The application of paclobutrazol resulted in a reduction in the amount of shattering losses. Our results were in line with earlier reported investigations where paclobutrazol application significantly reduced the shattering [28, 35, 50]. A higher shattering percentage affects the total yield of the sesame crop. As a result, the shattering percentage might have a considerable impact on crop production during harvest. The results show that employing paclobutrazol can dramatically lower shattering % while increasing yield. There was a significant difference in shattering percentage for paclobutrazol treatments. Highest shattering percentage was observed in the control while lowest was observed in P_5_ (600 mg L^−1^). PGRs and desiccants are used to manage the shattering losses and increase seed yield [33]. In the study, our results indicate that paclobutrazol significantly reduced the shattering losses in the sesame crop at harvesting.

Significant biomass increase in the sesame biological yield was observed by the five paclobutrazol application. Highest biomass was recorded for paclobutrazol treatments P_4_ (450 mg L^−1^), whereas the minimum in control. The possible reason for the increase in the biomass might be due to the increased pod photosynthetic rate in aboveground parts of the plants in the paclobutrazol treatment [35, 50]. However, the biomass partitioning among the vegetative and reproductive part varied due to the paclobutrazol retarding effects on the vegetative growth but it enhanced reproductive and root growth [71, 72]. Moreover, depending on the paclobutrazol concentration, nutrient utilization of plants also enhanced [73].

Grain yield considerably varied for the five paclobutrazol treatments. Moreover, for the paclobutrazol treatments the lowest grain yield was obtained in the control while the highest in the P_4_ (450 mg L^−1^). This increase in seed yield could be attributed to a variety of factors influenced by the paclobutrazol administration. The increased number of capsules per unit area and thousand grain weight that significantly influenced seed yield were the main contributor to the rise in seed yield following paclobutrazol treatments. In this study, findings were in consistent with earlier reported results by other researchers [35, 74].

The genetic pool and frequent recombination are the primary causes of phenotypic diversity [6]. On the PC1 and PC2 axes, the spatial representation of distances between genotypes revealed a considerable degree of phenotypic variation (Fig. 3). There were three potential genotypes PI – 175,902 (G_10_L_2_Y_2_), PI – 154,304 (G_2_L_2_Y_2_), and PI – 154,304 (G_2_L_2_Y_1_) with high scores in explaining phenotypic diversity: Furthermore, the fundamental features that revealed phenotypic diversity in this investigation were supported by Dubey et al. [75]. They concluded that SY, BIOY, TSW, NCP, PH, and NSC are significantly contributive features for estimating diversity that might be used as a selection criterion.

## Conclusions

It can be concluded that genotypes PI-175907, PI-154304 and PI-153509 produced the highest yield. These genotypes may be recommended for rainfed production in Pothwar since they fared well in shattering resistance and yield characteristics. Paclobutrazol significantly improved growth metrics, boosted yield, and reduced shattering losses in sesame genotypes. After 40 days of seeding, foliar treatment of paclobutrazol at 450 mgL^−1^ concentration significantly increased seed yield. The drop in sesame production in high rainfall locations was the least stable. As a result, low and medium rainfall areas are most suited for sesame production.

## Data Availability

All data generated or analysed during this study are included in this published article.
